# Detection and Genotyping of *Helicobacter pylori* among Gastric ulcer and Cancer Patients from Saudi Arabia

**DOI:** 10.12669/pjms.332.12024

**Published:** 2017

**Authors:** Fehmida Bibi, Sana Akhtar Alvi, Sara Ali Sawan, Muhammad Yasir, Ali Sawan, Asif A. Jiman-Fatani, Esam I. Azhar

**Affiliations:** 1 Fehmida Bibi, Special Infectious Agents Unit, King Fahd Medical Research Center, King Abdulaziz University, Jeddah, 21589, Kingdom of Saudi Arabia; 2 Sana Akhtar Alvi, Special Infectious Agents Unit, King Fahd Medical Research Center, King Abdulaziz University, Jeddah, 21589, Kingdom of Saudi Arabia; 3 Sara Ali Sawan, Department of Medicine and Surgery, Faculty of Medicine, King Abdulaziz University, Jeddah, 21589, Kingdom of Saudi Arabia; 4 Muhammad Yasir, Special Infectious Agents Unit, King Fahd Medical Research Center, King Abdulaziz University, Jeddah, 21589, Kingdom of Saudi Arabia; 5 Ali Sawan, Department of Anatomical Pathology, Faculty of Medicine, King Abdulaziz University, Jeddah, 21589, Kingdom of Saudi Arabia; 6 Asif A. Jiman-Fatani, Department of Medical Microbiology and Parasitology, Faculty of Medicine, King Abdulaziz University, Jeddah, 21589, Kingdom of Saudi Arabia; 7 Esam I. Azhar, Special Infectious Agents Unit, King Fahd Medical Research Center, King Abdulaziz University, Jeddah, 21589, Kingdom of Saudi Arabia

**Keywords:** *H. pylori*, Genotyping, Gastric ulcer, Gastric cancer

## Abstract

**Background and Objectives:**

*Helicobacter pylori* (*H. pylori*) infection is cause of several gastrointestinal diseases in humans. Virulence genes of *H. pylori* are associated with severity of disease and vary geographically. The aim of present study was to detect *H. pylori* in formalin-fixed paraffin-embedded (FFPE) tissues and further investigate prevalence of *babA_2,_ cag*A, *iceA1_,_ iceA2, vacA s1/s2* and *vacA m1/m2* genotypes in *H. pylori* from gastric cancer (GC) and gastric ulcer (GU) patients’ biopsy samples.

**Methods:**

We used FFPE tissues of 35 GC and 10 GU patients’ biopsy samples. Using Polymerase Chain Reaction (PCR), detection of *H. pylori* strain was performed by using specific primers targeting 16S rRNA and *ureC* encodes for phosphoglucosamine mutase genes. We have identified different virulence genes of *H. pylori* by PCR.

**Results:**

Of all the 45 samples tested, 20 GC and all 10 GU samples were positive for identification of *H. pylori* using specific genes (16S rRNA and *ureC*). The prevalence of *babA_2_*(100%) was significantly higher in GC as compared to GU (40%) samples. The rate of virulence genes *vacAs1* was higher in both GU 8 (80%) and GC (100%).

**Conclusions:**

Our study finds that *vacAs1am1* and *babA_2_* are most prominent genotypes and may play role in development of Gastric cancer.

## INTRODUCTION

*Helicobacter pylori* (*H. pylori*) is a pathogenic bacterium that inhabits gastric mucosa of humans and cause several gastrointestinal diseases including gastric cancer (GC). In developing countries prevalence of *H. pylori* infection may exceed as compared to developed countries where 20-50% are affected with infection.^1^ Previous studies suggest that *H. pylori* is genetically variable and certain genotypes are only detected in certain populations.^2^ Several virulence factors of *H. pylori* strain, such as *babA2*, *cagA*, *vacA*, and *iceA1* *have* been identified and involved in pathogenesis of infection.^3^

Recently reported blood group antigen-binding adhesin (*BabA*), a membrane protein of *H. pylori* help in binding to gastric epithelium. Three different *bab* alleles have been reported where only the *babA2* is functional for binding activity.^4^ Previous studies have reported prevalence of *babA2* positive strain in peptic ulcer and GC but with conflicting results.^5^ Therefore, relationship between *H. pylori* genotype and disease condition may vary from one geographic region to other.

The cytotoxin-associated gene A (*cagA*) is present in almost 50% of *H. pylori* strains and is constituent of genomic pathogenecity island (*cag*-PAI) responsible for type IV secretion system. The *cag*A-positive strains of *H. pylori* are responsible for mucosal inflammation and interleukin-8 (IL-8) production and are associated with pathogenesis of gastric cancer.^6^ In Asian countries, rate of *cag*A positivity has been reported in almost all strains of *H. pylori* isolated from infected cases.^7^

The vacuolating cytotoxin gene (*vac*A) encodes for vacuolating cytotoxin and found in all strains of *H. pylori*. It is involved in pathogenesis of peptic ulcer and GC by injuring gastric epithelial cells.^8^ All strains of *H. pylori* contain the *vac*A gene, and have two variable parts i.e signal region (*s1/s2*) and middle region (*m1/m2*). Previous studies have reported *vac*A allelic variations in different geographical regions as well as their toxic activity.^9^

Recently another virulence gene of *H. pylori*, *ice*A (the induced by contact with epithelium) has been reported. The *ice*A gene has two allelic types, *ice*A1 and *ice*A2 where *ice*A1 is significantly expressed and associated with peptic ulceration.^10^ The *ureC* encodes for phosphoglucosamine mutase, and renamed as *glmM* gene. The *ureC* (*glmM*) gene is easily detectable in *H. pylori* strains and involved in the development of the bacterial cell wall and growth.^11^

In Saudi Arabia, *H. pylori* infection rate is high in the Eastern, Central and Western region of the Kingdom. Previous studies have reported some virulence genes such as *cagA*, *iceA*1, and *iceA*2 in peptic ulcer and gastric ulcer in Saudi population.^12^,^13^ Detection of virulence genes of *H. pylori* mentioned above has not been reported yet in GC patients in Saudi Arabia. The genotyping of *H. pylori* is useful to determine epidemiological importance of *H. pylori* strain. Therefore, we designed a study to detect the presence of *H. pylori* using specific *glmM* and 16S rRNA. Further prevalence of virulence genes *babA_2,_* cagA, *iceA1_,_ iceA2, vacA s1/s2* and *vacA m1/m2* from paraffin-embedded (FFPE) gastric biopsies collected from GC and GU Saudi patients have been studied.

## METHODS

### Biopsy samples Collection and DNA extraction

Gastric FFPE biopsy specimens were collected from 35 GC patients and 10 with GU who had undergone gastric endoscopy in King Abdulaziz University (KAU) hospital in Jeddah, Saudi Arabia, between 2000-2014. Written informed consent was taken from all the patients. This study was approved by the Ethics and Research Committees of the hospital KAU. DNA was extracted from the biopsies using QIAamp DNA FFPE Tissue Kit (Qiagen, Hilden, Germany). Extracted DNA was further used for PCR.

### Polymerase Chain Reaction (PCR) assays

To detect *H. pylori* strain in biopsy samples from GC and GU patients specific primers targeting 16S rRNA and *ureA* genes were used. Further prevalence of virulence factors of *H. pylori* strain, specific primers targeting *babA_2,_* *cag*A, *iceA1_,_ iceA2, vacA s1/s2, vacA m1/m2* were used for PCR (Table-I). Amplification was conducted in a total volume of 25μL. The reaction mixture contained 13 μL, 2X ready PCR mix (Thermo Scientific), 2μL of each forward and reverse primers (Table-I), 1 ug DNA template, and 9μL nuclease free distilled water to a total volume of 25μL. The PCR amplification was performed according to the following program: an initial denaturation at 95°C for five minutes, followed by 35 cycles of denaturation at 95°C for 50s, annealing for 50s (Table-I), and a final extension at 72°C for five minutes. The amplified PCR products were electrophorsed (100 V/35 min) using 1% agarose gel. DNA ladder of 100 bp (Norgen, Canda) was used to determine the size of the amplified bands.

**Table-I:** Primers set and conditions used for PCR in this study.

*Genes*	*Nucleotide sequence (5'-3')*	*PCR product (bp)*	*PCR conditions*	*Reference*
16S rRNA	GCGACCTGCTGGAACATTAC CGTTAGCTGCATTACTGGAGA	138 bP	95ºC, 50 s; 60ºC, 50s; 72ºC, 50 s (35 cycles)	28
glmM	AAGCTTTTAGGGGTGTTAGGGGTTT AAGCTTACTTTCTAACACTAACGC	294 bp	95ºC, 50 s; 56ºC, 50s; 72ºC, 50 s (35 cycles)	23
babA2	CCAAACGAAACAAAAAGCGT GCTTGTGTAAAAGCCGTCGT	271 bp	95ºC, 50 s; 44ºC, 50s; 72ºC,50 s (35 cycles)	29
iceA1	GTGTTTTTAACCAAAGTATC CTATAGCCATTATCTTTGCA	247 bp	95ºC, 50 s; 57ºC, 50s; 72ºC,50 s (35 cycles)	29
iceA2	GTTGGGTATATCACAATTTAT TTTCCCTATTTTCTAGTAGGT	229 bp	95ºC, 50 s; 57ºC, 50s; 72ºC,50 s (35 cycles)	28
cagA	TTGACCAACAACCACAAACCGAAG CTTCCCTTAATTGCGAGATTCC	183 bp	95ºC, 50 s; 60ºC, 50s; 72ºC,50 s (35 cycles)	35
vacA s1/s2	ATGGAAATACAACAAACACAC CTGCTTGAATGCGCCAAAC	259/286 bp	95ºC, 50 s; 58ºC, 50s; 72ºC,50 s (35 cycles)	20
vacA m1/m2	CAATCTGTCCAATCAAGCGAG GCGTCAAAATAATTCCAAGG	567/642 bp	95ºC, 50 s; 54ºC, 50s; 72ºC,50 s (35 cycles)	14

### Statistical Analysis

Data of two groups were compared by using chi-square test using SPSS statistical software Version 9 (SPSS Inc., Chicago, IL, USA). A *P* value less <0.05 was considered as significant.

## RESULTS

This study included 45 FFPE gastric biopsy samples from Saudi patients. A total of 45 gastric patients, 13 (28.8%) females and 32 (71.2%) males ranging in age from 18 to 87 years, were included in the study. Among 45 cases, 35 (77.7%) were diagnosed as GC and 10 (22.3%) were GU cases (Table-II).

**Table-II:** Demographic characteristics and prevalence of virulence genes in different groups.

	*Group I (GU) (n=10)*	*Group II (GC) (n=35)*
Age: mean	31.3±7.5	58.2±14.6
***Gender:***		
Male	3 (30%)	29 (82.9%)
Female	7 (70%)	6 (17.1%)
babA2	4 (4%)	20 (100%)
iceA1	0 (0)	3 (15%)
iceA2	0 (0)	3 (15%)
cagA	3 (30%)	8 (40%)
vacA s1	5 (50%)	12 (60%)
vacA s2	0 (0)	0 (0)
vacA s1 s2	3 (30%)	8 (40%)
vacA m1	0 (0)	3 (15%)
vacA m2	2 (20%)	3 (15%)
vacA m1/m2	0 (0)	0 (0)

**Fig.1:**
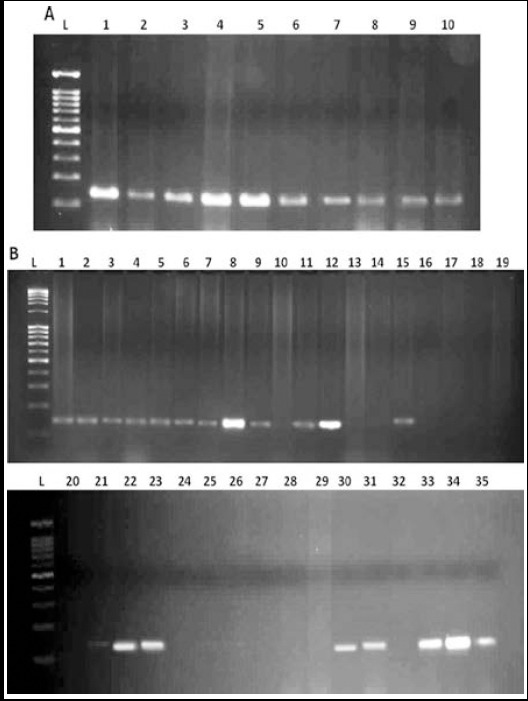
Detection of 16s rRNA gene. (A) Lane L, marker; 1-10, GU samples (B) Lane L, marker; 1-35, GC samples.

The detection of *H. pylori* was investigated using PCR. Among 45 FFPE gastric tissues, 20 (57.2%) GC and 10 (100%) GU biopsy samples were positive for *H. pylori* using 16S rRNA (Fig. 1a and b) and *glmM* genes. We examined six different *H. pylori* virulence genes in gastric biopsy samples. Among *H. pylori* positive gastric tissues prevalence of *babA_2_* gene was higher (100%) in GC tissues as compare to GU (4%). The presence of *cagA* gene yield a fragment of 183 bp using PCR. Amplification of *iceA1* and *iceA2* gene was performed using specific primers (Table-II). Prevalence of both *iceA1* and *iceA2* was detected only in three GC samples (15%) and was negative for GU samples. Only eight samples (40%) from GC and three (30%) from GU was positive for *cagA*. There was no significant difference for different genotypes among two different goups of patients (*P* > 0.05).

The detection of *vacA s1/s2* allele showed presence in 20 (100%) samples from GC and 8 (80%) from GU. The most virulent *vacA s1* was detected in most of the samples as it was detected in 12 (60%) samples from GC and 5 (50%) samples from GU. Whereas, *vacA s2* was not detected in any sample. Combined *vacA s1/s2* was detected in 8 (40%) GC and three (30%) GU samples. Detection of *vacA m1/m2* yielded fragment of 567/642 bp product. Only two (20%) samples were positive for *vacA m2* from GU samples. While for GC samples, allele *vacA m1* was detected in 3 (15%) samples and *vacA m2* was also detected in 3 (15%) samples. Combined *vacA m1/m2* was not detected in any sample (*P* > 0.05). In our study *vacA s1* was common allele in GC while *vacA s1/s2* was predominant genotype in GU samples (Table-II).

## DISCUSSION

Gastric cancer is an important cause of the death not only in Saudi Arabia but all over the world. Several previous studies have reported the importance of the *H. pylori* virulence genotypes. However, very few studies are available regarding *H. pylori* genotypes in Saudi population. Our study has confirmed high prevalence of *H. pylori* infection in GC and GU biopsy samples. In this study we have investigated the prevalence of various virulence factors (*babA_2,_* *cag*A, *iceA1_,_ iceA2, vacA s1/s2, vacA m1/m2*) from *H. pylori* positive GC and GU biopsy samples using PCR. Prevalence of *H. pylori* and Gastric Cancer is high in Asian countries such as Japan and Korea as compared to other countries.^14^ In this study we have seen high prevalence of *H. pylori* virulence factors in GC and GU samples. Different genes contribute in colonization of *H. pylori* to gastric epithelium of humans.^15^ The *glmM* and 16S rRNA gene has more sensitivity than other genes for detection in gastric biopsies samples.^16^ Therefore, we have used *glmM* and 16S rRNA for detection of *H. pylori* in biopsy samples.

Virulence genes of *H. pylori* *cag*A and *vac*A are important adherence factors involved in gastrointestinal diseases.^17^-^19^ In our study we found low frequency of *cag*A in both groups. cagA is associated with the development of gastric carcinoma and it is an important marker for the most virulent strains associated with gastric severe infection.^20^ Previous studies have shown relationship of gastric cancer and *cagA* positive *H. pylori*^21^ while other show contradictory results like we have in our study. We have used *Vac*As1/s2 and *Vac*Am1/m2 type virulence factor for detection and according to our results prevalence of *Vac*As1 is high in both GU and GC. *Vac*As1 genotype is associated with high toxin activity and severe diseases. Previous study from China has also reported similar results.^22^

The *babA_2_* gene has been shown to be associated with high risk of gastric cancer and also has strong relation with *Vac*As1.^8^ In GC samples we have found high prevalence of *babA_2_* and *Vac*As1. A previous study has reported similar results where these genotypes were associated with increased risk of developing cancer.^23^

*H. pylori* *iceA1* positive strains are associated with peptic ulcers due to the production of IL-8 produce by these strains.^24^ In our study prevalence of *iceA1* and *iceA2* is lower in both GC and GU samples. Our study is consistent with some previous studies^25^ while contradictory results are found in other.^26^ Therefore, the both alleles only detected in GC samples but not in GU. The *babA2* gene of *H. pylori* is an important contributor for higher risk of GU and GC development. Our results are similar to previous study where *H. pylori* strains carrying *babA2*, *cagA*, and *vacAs1* genotypes were associated with the risk of intestinal cancer.^8^ Several virulence factors contribute in the pathogenesis of *H. pylori* have been studied here.

## CONCLUSIONS

Our study report first time high incidence of *H. pylori* virulence genes in Saudi patients with GC and GU. Prevalence of cagA genotype is not associated with severity of disease and *iceA1* allele was detected in GC patients only. We have seen in our study high frequency of genes *babA_2_* and *VacA*s1 in GC patients.

### Authors’ Contributions

**FB** made substantial contributions to design this study.

**SA, MY, and EIA** were involved in PCR and data interpretation.

**AJF, SAS, and AS** were responsible for sample collection and clinical databases.

**FB** drafted the manuscript.

All authors have read and approved the final manuscript.

## References

[1] Miwa H, Go MF, Sato N (2002). H. pylori and gastric cancer:the Asian enigma. Am J Gastroenterol.

[2] Sicinschi LA, Correa P, Peek RM, Camargo MC, Delgado A, Piazuelo MB (2008). *Helicobacter pylori* genotyping and sequencing using paraffin-embedded biopsies from residents of Colombian areas with contrasting gastric cancer risks. Helicobacter.

[3] Erzin Y, Koksal V, Altun S, Dobrucali A, Aslan M, Erdamar S (2006). Prevalence of *Helicobacter pylori* vacA, cagA, cagE, iceA, babA2 genotypes and correlation with clinical outcome in Turkish patients with dyspepsia. Helicobacter.

[4] Pride DT, Meinersmann RJ, Blaser MJ (2001). Allelic variation with in *Helicobacter pylori babA* and *babB*Infect. Immun.

[5] Mattar R, dos Santos AF, Eisig JN, Rodrigues TN, Silva FM, Lupinacci RM (2005). No correlation of babA2 with vacA and cagA genotypes of *Helicobacter pylori* and grading of gastritis from peptic ulcer disease patients in Brazil. Helicobacter.

[6] Van der Ende A, Pan ZJ, Bart A (1998). *cagA*-positive *Helicobacter pylori* populations in China and the Netherlands are distinct. Infect Immun.

[7] Ito Y, Azuma T, Ito S (1997). Analysis and typing of the *vacA* gene from *cagA*-positive strains of *Helicobacter pylori* isolated in Japan. J Clin Microbiol.

[8] Chomvarin C, Namwat W, Chaicumpar K, Mairiang P, Angchan A, Ripa B (2008). Prevalence of Helicobacter pylori vacA, cagA, cagE, iceA and babA2 genotypes in Thai dyspeptic patients. Int J Infect Dis.

[9] Atherton JC, Cao P, Peek RM (1995). Mosaicism in vacuolating cytotoxin alleles of *Helicobacter pylori* or Association of specific *vacA* types with cytotoxin production and peptic ulceration. J Biol Chem.

[10] Peek RM, Thompson SA, Donahue JP, Tham KT, Atherton JC, Blaser MJ (1998). Adherence to gastric epithelial cells induces expression of a *Helicobacter pylori* gene *iceA* that is associated with clinical outcome. Proc Assoc Am Physicians.

[11] Espinoza MGC, Vazquez RG, Mendez IM, Vargas CR, Cerezo SG (2011). Detection of the glmM gene in Helicobacter pylori isolates with a novel primer by PCR. J Clin Microbiol.

[12] BinSaeed AA (2009). Glimpse of the epidemiological research on Helicobacter pylori in Saudi Arabia. Saudi J Gastroenterol.

[13] Kadi RH, Halawani EM, Abdelkader HS (2014). Prevalence of H.pylori strains harbouring cagA and iceA virulence genes in Saudi patients with gastritis and peptic ulcer disease. Microbiology Discovery.

[14] World Cancer Report 2014 (2014). World Health Organization.

[15] Wang G, Maier RJ (2009). A RecB-like helicase in *Helicobacter pylori* is important for DNA repair and host colonization. Infect Immunity.

[16] De Reuse H, Labigne A, Mengin-Lecreulx D (1997). The *Helicobacter pylori* ureC gene codes for a phosphoglucosamine mutase. J Bacteriol.

[17] Tanih NF, McMillan M, Naidoo N, Ndip LM, Weaver LT, Ndip RN (2010). Prevalence of *Helicobacter pylori* vacA, cagA and iceA genotypes in South African patients with upper gastrointestinal diseases. Acta Tropica.

[18] Miehlike S, Schuppler M, Frings C (2001). *Helicobacter pylori* vacA, iceA and *cagA* status and pattern of gastritis in patients with malignant and benign gastroduodenal disease. Am J Gastroenterol.

[19] Yamaoka Y, Kodama T, Gutierrez O, Kim JG, Kashima K, Graham DY (1999). Relationship between *Helicobacter pylori iceA*
*cagA* and *vacA* status and clinical outcome:studies in four different countries. J Clin Microbiol.

[20] Chih-Ho L, Ju-Chun H, Chiang-Ni C, Ju-Pi L, Lii-Tzu W, Hua-Shan W (2014). Mixed Infections of Helicobacter pylori Isolated from Patients with Gastrointestinal Diseases in Taiwan. Gastroenterol Res Pract.

[21] Tiwari SK, Manoj G, Kumar GV, Sivaram G, Hassan SI, Prabhakar B (2008). Prognostic significance of genotyping *Helicobacter pylori* infection in patients in younger age groups with gastric cancer. Postgrad Med J.

[22] Aziz F, Chen X, Yang X, Yan Q (2014). Prevalence and correlation with clinical diseases of *Helicobacter pylori* cagA and vacA genotype among gastric patients from Northeast China. BioMed Research international.

[23] Höcker M, Hohenberger P (2005). *Helicobacter pylori* virulence factors—one part of a big picture. Lancet.

[24] Al Qabandi A, Mustsfa AS, Siddique I, Khajah AK, Madda JP, Junaid TA (2005). Distribution of *vacA* and *cagA* genotypes of *Helicobacter pylori* in Kuwait. Acta Trop **.

[25] Lin HJ, Perng CL, Lo WC, Wu CW, Tseng GY, Li AFY (2004). *Helicobacter pylori* cagA, iceA and vacA genotypes in patients with gastric cancer in Taiwan. World J Gastroenterol.

[26] Smith SI, Kirsch C, Oyedeji KS, Arigbabu AO, Coker AO, Bayerdöffer E (2002). Prevalence of *Helicobacter pylori* vacA, cagA and iceA genotypes in Nigerian patients with duodenal ulcer disease. J Med Microbiol.

